# Ethical issues in preconception genetic carrier screening

**DOI:** 10.1080/03009734.2016.1189470

**Published:** 2016-07-07

**Authors:** Ulrik Kihlbom

**Affiliations:** Center for Research Ethics and Bioethics, Uppsala University, Uppsala, Sweden

**Keywords:** Ethics, justice, medicalization, preconception genetic carrier testing, reproductive autonomy

## Abstract

Population-based preconception genetic carrier screening programmes (PCS) with expanded panels are currently being developed in the Netherlands. This form of genetic screening for recessive traits differs from other forms of genetic testing and screening in that it is offered to persons not known to have an increased risk of being carriers of genetic traits for severe recessive diseases and in that they include tests for a large number of traits, potentially several hundred. This raises several ethical issues around justice, consequences, and autonomy. It will be argued that most of these ethical problems call for cautious reflection when setting up PCS and similar programmes within preconception care. It is moreover argued that it is ethically problematic to have an official aim and failing to mention possibly legitimate public aims that actually drive the development of PCS.

## Introduction

This article introduces and discusses some pertinent ethical issues of population-based preconception genetic carrier screening programmes (PCS). The aim is to provide a conceptual framework that may enhance ethical debate and reflection of preconception care in general and PCS in particular. After a brief description of PCS, three ethical grounds for the widely accepted criteria for screening programmes are distinguished, and a number of issues are identified under these headings.

Preconception genetic carrier testing has for some years targeted groups, families, or individuals, such as the Ashkenazi Jews and the population at Cyprus, who have a known increased risk for being carriers of severe recessive autosomal diseases (1). In these cases, couples belonging to such subpopulations are tested for single or a few recessive traits, and there are no longer any major ethical controversies about whether or not to have the screening programmes. The PCS that are currently being developed in the Netherlands differ from earlier screening programmes in two ways (2). Firstly, they target couples wanting to have a child and without known increased risk for recessive autosomal diseases, i.e. the general population. Secondly, these programmes test individuals for a large number of traits. PCS is not yet in practice in European health care even though available through commercial companies. Pilot programmes are currently being developed in at least two university hospitals in the Netherlands. The PCS programme being developed at the university hospital in Groningen includes a panel of 50 recessive traits for severe and rare diseases with early onset (2). Potentially, test panels can be further expanded to several hundred recessive traits. Couples for whom the results of the test show that both partners are carriers of such a trait have different options when it comes to family planning. They can decide to live with the 25% risk of having a child with the disease and choose not to do anything. Options to avoid the risk range from non-medical options like refraining from having children, adopting children, or even changing partner, to the use of reproductive technologies like *in vitro* fertilization (IVF) and preimplantation diagnostics, prenatal diagnostics, or sperm/egg donation.

## Discussion

Currently, there are no medical consensus statements or professional society guidelines regarding the use of PCS. If and when such guidelines are put into place, one might expect that they will not diverge much from the criteria used in order to assess other forms of screening programmes. Wilson and Jungner proposed 10 criteria for screening programmes almost 50 years ago (3). A more contemporary set of criteria adapted to genetic screening, suggested by Andermann, is the following (4):

1. The screening programme should respond to a recognized need.
2. The objectives of screening should be defined at the outset.
3. There should be a defined target population.
4. There should be scientific evidence of screening programme effectiveness.
5. The programme should integrate education, testing, clinical services and programme management.
6. There should be quality assurance, with mechanisms to minimize potential risks of screening.
7. The programme should ensure informed choice, confidentiality and respect for autonomy.
8. The programme should promote equity and access to screening for the entire target population.
9. Programme evaluation should be planned from the outset.
10. The overall benefits of screening should outweigh the harm. (4)

Exactly how these criteria should be interpreted and weighted against each other must, presumably, be a question to be answered in each particular case. The ethical grounds that reasonably justify having these screening criteria rather than others can be summarized as three broad normative ideas:

- Justice
- Consequences
- Autonomy

Considerations of justice seem clearly to be behind criterion 8, but can also be viewed as the basis of criteria 4 and 5 as effectiveness in health care is important for the capacity to allocate recourses in a fair way. The appeal to consequences justifies primarily criteria 1, 3–6, 9, and 10. Thus, in the case of screening programmes, the consequences that may provide good reasons for implementation concern satisfying needs in terms of promotion and maintenance of health and well-being, avoidance and reduction of suffering, and keeping societal costs low in an effective and good way. Criterion 7 obviously concerns respect for and promotion of autonomy.

The ethical discussion on PCS and reproductive ethics circles around one or several of these three normative ideas that are three of the most central ideas in ethics. The following is not intended as a full list or exploration of ethical issues that can be related to PCS. The vast discussion on genetic results and integrity, abortion, or duties to future generations will be omitted. Some of the issues will only be mentioned below; the few discussed in some detail are those less discussed in the literature and most relevant for PCS and preconception care in general.

### Justice

One of the debates related to justice is the issue of prioritization. How should resources within preconception care be prioritized in relation to other health care resources (5–8)?

Another issue of justice is that of discrimination and stigmatization, both with regard to those who will test positive as carriers, and with regard to people who will actually develop the disease that the screening programme tests for. Some authors highlight the risk that carriers of a recessive trait can have loss of self-worth and be subject of stigmatization (9–11). A more extensive ethical debate concerns the so-called *expressivist argument*. This argument claims that a preconceptional or prenatal measure that can be used to avoid the birth of children with certain traits expresses negative views to and about people with those traits (12–15). The expressivist argument and concerns for discrimination are obviously more relevant to PCS with test panels that include traits for diseases that allow a person to live a life of some length and quality. There are, surely, very severe early-onset recessive diseases that are incompatible with living with some quality of life. However, if the test panels were expanded so that they also include disease traits linked with variable penetrance, late onset, and substantial quality of life, then such PCS programmes certainly face this objection.

### Consequences

The objections concerning discrimination and stigmatization also relate to the possible consequences of PCS, the argument saying that there is a risk that screening programmes like PCS lead to discrimination, stigmatization, and even eugenics (15). The main problem of evaluating these kinds of ‘slippery slope’ arguments is not their *relevance*—discrimination, stigmatization, and even eugenics are bad consequences—but their *plausibility*. There is little evidence provided in the literature that there is a significant risk that something like PCS would lead to, for example, stigmatization.

A related concern regarding PCS and preconception care in general is that these practices lead to an increased *medicalization* of social life (16). Medicalization is a term with several usages in the literature. It was introduced in sociology to capture social processes in which medical concepts such as illness and health are applied to aspects of social life previously understood as being outside the realm of medicine (17). It was argued that medicalization of socially deviant behaviour, such as homosexuality and different forms of addiction, increased social control and thereby also individualized the problems that otherwise might be regarded as social. Medicalization has since then also been used to denote more normal parts of human life such as child-birth, ageing, and death, as well as more preventive areas of medicine and public health. The last-mentioned is highly relevant to PCS and to preconception care in general, the idea being that the amplified social emphasis on health, in an increasing number of aspects of life, makes people live their lives in accordance with prescriptions from health care professionals. In sociology, the concept of medicalization is used as a way to criticize these social processes (although it should be pointed out that these critics do not think that this is something intended by health care professionals, and neither do they identify health care as the main driving force of medicalization). However, a simple answer to the question whether or not medicalization is desirable seems not to be available. The medicalization of birth, for instance, has had many good consequences in terms of saved lives and reduced suffering. There might also be some bad consequences involved, but an evaluation of medicalization processes must be contextualized and empirically well informed. In the case of PCS and preconception care, it would be most interesting (albeit requiring a lot of effort) to have more thorough empirical prospective studies of the way in which such practices influence how people perceive the planning of pregnancy and the making of a family.

### Autonomy

A concept related to medicalization is that of *routinization*. This concept has been especially discussed in the context of reproductive medicine and in relation to informed choices and autonomy. That a practice becomes routine within medicine and is considered as standard medical care seems to affect the normative perceptions people have regarding that practice (18,19). A well-known example is that of ultrasound screening including tests for trisomy chromosomal disorders that has become routine in many countries. In these countries most women undergo ultrasound testing, and studies indicate that many women think of such tests as ‘the responsible’ choice that also ‘protects the health’ of their child, although abortion is the only alternative to having the child (20,21). This has led these authors and many others to question the extent to which women have an autonomous choice in these contexts. The implementation of PCS could lead to a similar form of criticism. Also here it should be pointed out that health care it is not necessarily the driving force behind the shift in normative expectations. Couples and their social environment may well be essential factors in this development, which implies that any effort to adjust should take into account health care professionals and patients as well as their partners.

The nature of PCS adds further complexity with regard to informed choice and autonomy. With expanded test panels, the risk estimation will become quite hard both for couples and for those health care professionals counselling them. Many of the traits tested for are very rare but also very severe, so the weighing of the severity of the outcome versus its probability will be difficult. Moreover, reduced penetrance and variable expressivity may bring further uncertainty into the decision-making matrix. For example, for some recessive traits, not all individuals with the genetic trait will develop the features of the disease (reduced penetrance), and those features may be different for different individuals (variable expressivity). Furthermore, with an increased number of traits on the test panel, there might be more than one disease to consider. These are not necessarily insurmountable problems, but they will require a cautious and reflective development of the decision-making procedures around PCS.

### Autonomy or consequences and autonomy—what are the legitimate aims of PCS?

Related to both the ethical notion of consequences and that of autonomy is the debate concerning the aims of reproductive screening programmes. In fact, the debate can be said to concern the question whether reproductive autonomy should be the primary goal or whether a more pluralistic account including public health considerations is a proper aim. The pluralist aim would include reproductive autonomy as well as improving population health by reducing the prevalence of disability and disease in the newborn population, and reducing future health and social welfare costs. The most cherished aim of screening programmes within reproductive health care is no doubt enhancement of reproductive autonomy (22–25). For instance, de Jong et al. say, when discussing prenatal genetic screening, that:

Enabling meaningful reproductive choice with regard to parenting or avoiding a child with a serious disorder or disability is (or should be) the very aim of offering testing for fetal abnormalities. (22)

Specifically discussing PCS, De Wert et al. claim that:

[T]here are good moral reasons for regarding the enhancement of reproductive autonomy rather than prevention as the primary objective both of individual preconception genetic counselling and of PCS. (23)

There are a number of problems with this view. First, public health considerations are in fact among the actual motives for having PCS programmes and preconception care in general. Second, such motives can be perfectly legitimate aims for a carrier screening. Third, there is no necessary opposition between public health aims and reproductive autonomy. Finally, there is the corollary problem of having an official goal and other actual goals of the preconception programmes. These problems will be addressed in order.

It seems quite obvious that public health considerations are among the actual motives for developing PCS. Non-medical indications for offering PCS are not in question so far. The traits considered for being included in PCS are all recessive traits for severe diseases. Having recessive traits such as, for example, height and eye size on the test panel would arguably enhance reproductive options, but no one has seriously proposed such a test panel, and for good reasons. Neither is anyone questioning the criteria put forward by Andermann stating that: ‘there must be a proven positive balance of benefits over harms for those participating’ (4). The harms and benefits considered regarding this criterion must, arguably, also include suffering and well-being of possible future beings. To avoid suffering, pressure, anxiety of couples, and costs of society seems, hence, to be actual motives behind PCS, as well as for other public health interventions.

Such aims can also be perfectly reasonable for preconception screening programmes, as well as the aim of promoting reproductive autonomy—at least within publicly funded health care systems where it seems simply unethical not to consider societal costs, how other patient groups are affected, and the general public health. The main worry of bringing in societal concerns among aims of PCS and other reproductive screening programmes is that this would pave the way for eugenics:

Promoting informed choice is commonly recognized as the chief purpose and benefit of prenatal screening, its very presence being viewed as a key way in which the process can be distanced from eugenics. (23)

However, as others have pointed out, eugenics does not necessarily issue from the state, nor involve coercion (26,27). In a liberal society where PCS has become a routine, and if social norm pressure was put on couples to make certain choices, informed choice is no guarantee against eugenics. Through the routinization of PCS, couples may not really consider this as being offered an additional reproductive choice but at as a standard part in preconception care.

Moreover, public health aims do not stand in any necessary opposition to the promotion of reproductive autonomy—quite the contrary. If the public health motives for setting up the PCS programme were openly declared together with a clearly expressed offer to make informed choices, then couples would have a set of values to contrast their own values against and form their own view. It is presumably easier for a couple to assume responsibility regarding their choice if it is clear that also health care professionals assume responsibility regarding their values and aims. To state public health aims openly is not the same as being directive in the sense of telling people what to do.

Lastly, there are several ethical problems with having an official aim and another set of actual aims that drive preconception care. Health care runs the risk of being accused of deception, which in turn may lead to lack of trust in preconception care. Furthermore, it constrains public debate by making it harder to debate the legitimacy of the aims. Suppose, for instance, that there were economic incentives for university hospitals for developing PCS programmes. The legitimacy of such incentives cannot be ruled out a priori, but such motives need to be discussed openly.

In conclusion, PCS raises several ethical issues that call for reflection on how PCS programmes should be implemented. There are issues around justice that concern prioritization, discrimination, and stigmatization. Among the relevant consequences of PCS, medicalization is of primary interest. If PCS becomes established as a part of basic health care, some aspects of private life will become part of health care, and that needs to be discussed by the parties involved. Issues around autonomy and informed consent are central in the discussion of PCS, and the phenomenon of routinization needs to be considered.

Most of these problems seem not to constitute decisive reasons against PCS, but are highly relevant to how the screening programme is set up and how the consent process is designed. However, a clear ethical problem is to have a discrepancy between an official aim and what really drives the development.
